# Clinical establishment of a laboratory developed quantitative HDV PCR assay on the cobas6800 high-throughput system

**DOI:** 10.1016/j.jhepr.2021.100356

**Published:** 2021-08-28

**Authors:** Lisa Sophie Pflüger, Dominik Nörz, Tassilo Volz, Katja Giersch, Annika Giese, Nora Goldmann, Dieter Glebe, Jan-Hendrik Bockmann, Susanne Pfefferle, Maura Dandri, Julian Schulze zur Wiesch, Marc Lütgehetmann

**Affiliations:** 1 Center for Diagnostics, Institute of Medical Microbiology, Virology and Hygiene, University Medical Center Hamburg-Eppendorf (UKE), Hamburg, Germany; 2 I. Department of Internal Medicine, University Medical Center Hamburg-Eppendorf (UKE), Hamburg, Germany; 3 Institute of Medical Virology, National Reference Centre for Hepatitis B Viruses and Hepatitis D Viruses, Justus Liebig University Giessen, Giessen, Germany; 4 German Center for Infection Research (DZIF), Giessen-Marburg-Langen, Germany; 5 German Center for Infection Research (DZIF), Hamburg-Lübeck-Borstel-Riems, Germany

**Keywords:** HDV, Hepatitis delta virus, RT-qPCR, Real time reverse transcription polymerase chain reaction, cobas6800, molecular diagnostics, viral hepatitis, quantification, CE-IVD, CE-marked *in vitro* diagnostics, cHDV, chronic HDV infection, EQA, external quality assessment, GT, genotypes, HDV_UCT, HDV utility-channel, LLOD, lower limit of detection, RT-qPCR, reverse transcription quantitative real-time PCR, WHO, world health organization

## Abstract

**Background & Aims:**

Currently available HDV PCR assays are characterized by considerable run-to-run and inter-laboratory variability. Hence, we established a quantitative reverse transcription real-time PCR (RT-qPCR) assay on the open channel of a fully automated PCR platform (cobas6800, Roche) offering improved consistency and reliability.

**Methods:**

A primer/probe-set targeting a highly conserved region upstream of the HDV antigen was adapted for use on the cobas6800. The lower limit of detection (LLOD) was determined using a dilution panel of the HDV WHO standard (n = 21/dilution). Linearity and inclusivity were tested by preparing 10-fold dilution series of cell culture-derived virus (genotype [GT]1-8; n = 5/dilution). Patient samples containing a variety of bloodborne viral pathogens were tested to confirm exclusivity (n = 60).

**Results:**

The LLOD of the HDV utility-channel (HDV_UTC) assay was determined as 3.86 IU/ml (95% CI 2.95–5.05 IU/ml) with a linear range from 10–10ˆ8 IU/ml (GT1). Linear relationships were observed for all HDV GTs with slopes ranging from -3.481 to -4.134 cycles/log and R^2^ from 0.918 to 0.994. Inter-run and intra-run variability were 0.3 and 0.6 Ct (3xLLOD), respectively. No false-positive results were observed. To evaluate clinical performance, 110 serum samples of anti-HDV-Ab+ patients were analyzed using the HDV_UTC and CE-IVD RoboGene assays. 58/110 and 49/110 samples were concordant positive or negative, respectively (overall agreement 97.3%). Quantitative comparison demonstrated a strong correlation (R^2^ 0.8733; 95% CI 0.8914–0.9609; *p* value <0.0001).

**Conclusion:**

The use of highly automated, sample-to-result solutions for molecular diagnostics holds many inherent benefits over manual workflows, including improved reliability, reproducibility and dynamic scaling of testing capacity. The assay we established showed excellent analytical and clinical performance, with inclusivity for all HDV GTs and a limit of quantification of 10 IU/ml, making it a sensitive new tool for HDV screening and viral load monitoring.

**Lay summary:**

The hepatitis delta virus (HDV) causes a severe form of inflammation in the liver. We developed a tool for molecular diagnostics, a polymerase chain reaction HDV assay that showed great performance. It can be used to improve diagnosis of HDV, as well as for monitoring treatment responses. The assay allows for quantification of the virus in the tested samples and is performed on a fully automated platform (cobas6800), which provides various benefits including less hands-on time and excellent comparability of test results.

## Highlights

• Establishment and validation of new quantitative HDV PCR assay on a fully automated platform (cobas6800).
• New assay allows for dynamic scaling of testing capacity and drastically reduces hands-on time as wells as manual steps.
• Improved reliability and reproducibility of test results.
• Lower limit of quantification of 10 IU/ml and a linear range from 10^1^–10^8^ IU/ml.
• Inclusivity for all 8 known HDV genotypes.

## Graphical abstract

**Figure:**
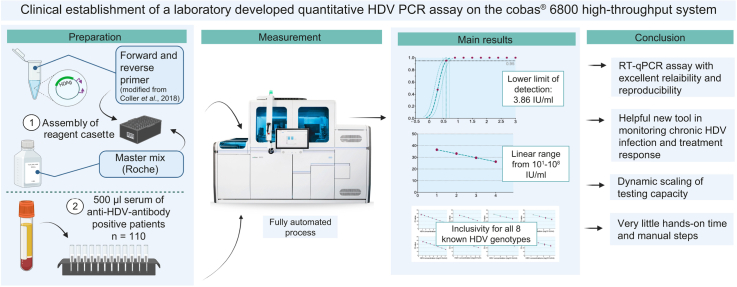

## Introduction

Chronic HDV infection (cHDV) is considered the most severe form of viral hepatitis. Studies have shown that high HDV viral loads (>600,000 copies/ml) in non-cirrhotic patients are associated with a higher risk of developing cirrhosis,^1^ which combined with the subsequently elevated risk of developing hepatocellular carcinoma and liver decompensation,^2^^,^^3^ contributes to the high morbidity and mortality associated with cHDV.^4^ Moreover, therapeutic options for cHDV are very limited and mainly based on PEGylated interferon alpha. Further, treatment is associated with low response rates (17–47%)^5^ and relapse after therapy.^6^ However, only in 2020, a synthetic polypeptide derived from the envelope protein of HBV (bulevirtide) was approved as a new treatment option by the European Medicines Agency. The drug inhibits the HBV/HDV entry receptor and was shown to induce a decrease of HDV RNA and normalization of alanine aminotransferase levels^7^ opening new therapeutic avenues, but also increasing the clinical need for tools to monitor HDV viral loads and hence treatment responses. On the molecular level, HDV is a negative strand, circular RNA virus with strong self-base-pairing and high genomic diversity between the different genotypes (GT).^8^^,^^9^ Both of these features pose a considerable challenge for the diagnostic workflow. Further, many available reverse transcription real-time PCR (RT-qPCR) assays are characterized by considerable run-to-run and inter-laboratory variability. Herein, we describe the establishment of a quantitative RT-qPCR assay on the open channel of a fully automated PCR-platform (cobas6800, Roche).

## Materials and methods

A primer/probe-set targeting a highly conserved region upstream of the HDV antigen coding region^10^ was selected and adapted for use on the cobas6800. Primers were modified with 2’-O-methyl bases in their penultimate base to prevent formation of primer dimers and the probe was conjugated to a minor groove binder at the 3’end in order to increase melting temperature and binding stability. Primers and probes were ordered from IDT DNA Technologies (Coralville, USA) and biomers.net GmbH (Ulm, Germany), respectively. Forward primer (5’-CTCCCTTWGCCATCCmGAG -3’; 208 μl; 1000 nM), reverse primer (5’-CTCTTCGGGTCGGCATmGG-3’; 208 μl; 1000 nM), probe (5’-ATGCCCAGGTCGGACCRC5-3’; 10.4 μl; 50 nM) and Utility Channel Master Mix Reagent 2 (UC MMX-R2, 5573.6 μl; Roche) were combined and transferred to a reagent cassette (96 reactions/cassette). Sequence-specific forward and reverse primers allow for selective amplification of the internal control (included in the UC MMX-R2). The temperature profile and Utility Channel software settings included a predefined uracil-DNA N-glycosylase incubation step, a pre-PCR step (1 cycle: 55°C for 120 s, 60°C for 360 s, 65°C for 240 s), 5 cycles of 95°C for 5 s and 55°C for 30 s (1^st^ measurement at the end of each cycle) followed by 45 cycles of 91°C for 5 s and 58°C for 25 s (2^nd^ measurement at the end of each cycle) and finished with a predefined cooling step. The minimum of the relative fluorescence increase was set at 1.25.

The lower limit of detection (LLOD) was determined using a 2-fold dilution panel of the HDV world health organization (WHO) standard (PEI-code: 7657/12; n = 21/dilution,^11^), which included 10 different concentrations, with the highest and lowest concentrations being 1,000 IU/ml and 1.95 IU/ml, respectively, and a set of 21 negative samples. Linearity and inclusivity were tested by preparing 10-fold dilution series of cell culture-derived virus (GT1-8; n = 5/dilution). To determine inter- and intra-run variability, the dilution panel used to assess LOD was split into groups of 7 samples/dilution and tested in different runs. Bloodborne viral pathogens for assessing exclusivity included HBV, HCV, EBV, HIV, BKV and CMV (n = 10 each).

Evaluation of the performance of the HDV utility-channel (HDV_UCT) assay included 20 samples of an external quality assessment (EQA) panel for HDV (INSTAND, Düsseldorf, Germany) and serum samples from HDV RNA-positive patients (n = 4) collected at different timepoints. To further assess assay performance and whether HDV PCR-positive patients have been missed due to a lack of sensitivity of the testing method currently in use, the clinical records of admitted patients at our center from 2008 until July of 2020 were screened for anti-HDV-antibody status. In total, 170 HDV patients were identified of whom 60 had to be excluded due to a lack of sample material. Of the remaining 110 patients, 1 serum sample each was included in this study. According to the records, 52.7% (58/110) of patients were HDV PCR positive at the time of blood sampling. Serum samples were stored at or below -18° Celsius until use. Samples containing less than 2,000 μl were diluted using negative human plasma (quantitative HBsAg dilution reagent, Abbott) to a total volume of 2,000 μl. All 110 samples were subjected to the new HDV_UCT assay and an already established CE-marked *in vitro* diagnostics (CE-IVD) assay (Analytik Jena, Jena, Germany). For the HDV_UCT assays, 500 μl of the sample was used. For the CE-IVD assay, 400 μl were utilized using the INSTANT Virus RNA/DNA Kit (Analytik Jena, Jena, Germany) to extract RNA before administering 5 μl of the eluate following the manufacturer’s recommendations. The nucleic acid extraction and RT-qPCR were carried out according to the manufacturer’s instructions and the latter was performed on a LightCycler 480 II (Roche, Rotkreuz, Switzerland).

The study was approved by the local ethics committee (PV5626). GraphPad Prism version 8 (San Diego, California, USA) was used for statistical analysis. To determine the LLOD a Probit analysis was computed.^12^ The graphical abstract was created with BioRender.com.

## Results

**Fig. 1:**
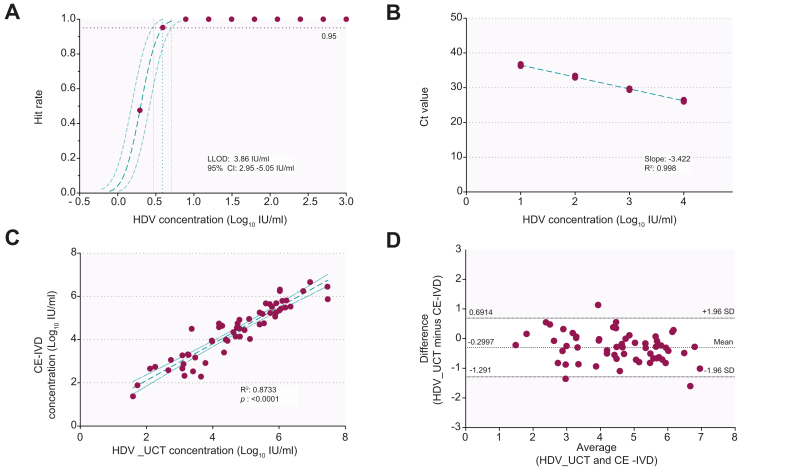
Probit analysis and linearity for HDV genotype 1 and test results of the new HDV_UCT assay compared to the CE-trademarked IVD RoboGene assay. (A) A Probit analysis was computed based on the results of a 2-fold dilution panel of the WHO standard (n = 21 repeats/dilution). The plot is displaying the calculated LOD of 3.86 IU/ml (dotted green line) and the corresponding 95% CI 2.95–5.05 (dotted light green line) as well as the Probit curve (green dashed line; 95% CI: light dashed green lines). The observed hit rates are marked by violet dots. (B) Displayed are the results (violet dots) of a 10-fold dilution panel of the WHO standard (GT1; n = 5 repeats/dilution). The dashed green line shows the linear regression line with a slope of -3.422 (R^2^ 0.998). (C) Comparison between the test results (violet dots) of the HDV_UCT and the CE-IVD assays demonstrated a strong correlation (R^2^ 0.8733; 95% CI 0.8914–0.9609; *p* value <0.0001). The linear regression line is plotted as a dashed green line (95% CI: light green dashed lines). Values are log-transformed. (D) Bland-Altman analysis based on the log-transformed test result (violet dots) in IU/ml of the HDV_UCT and the CE-IVD assays. The mean of the bias was calculated as -0.2997 (95% CI 0.6914 to -1.291). Only 3 data points were detected lying outside of the margins of ± 1.96 SD. CE-IVD, CE-marked in vitro diagnostics assay; HDV_UCT, HDV utility-channel; LLOD, lower limit of detection; WHO, world health organization.

**Fig. 2:**
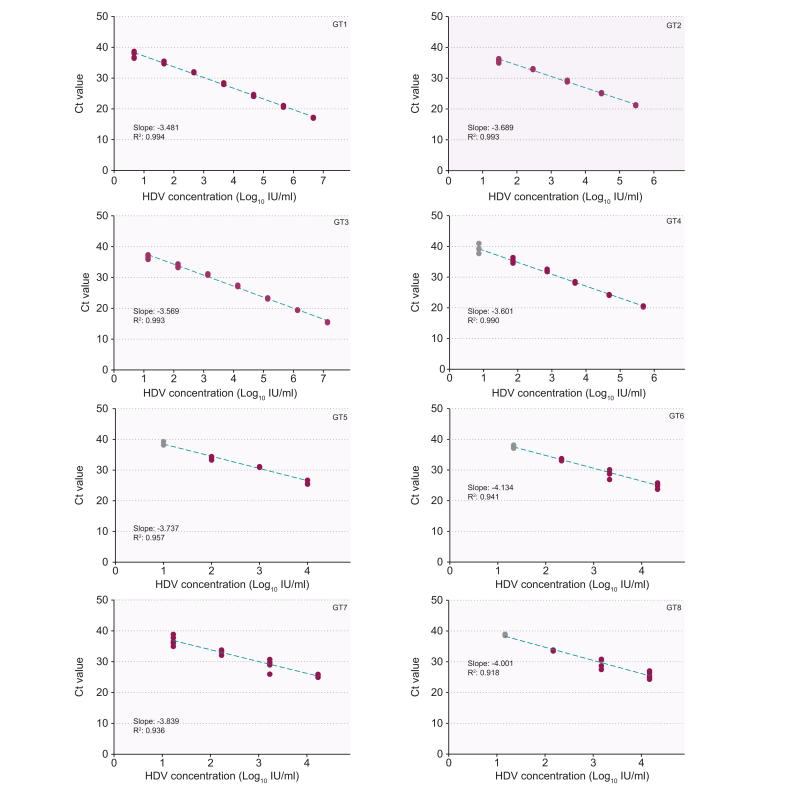
Linearity and inclusivity of cell culture-derived HDV genotypes 1-8. A linear relationship was observed for all 8 genotypes with slopes ranging from -3.481 to -4.134. The linear regression line is plotted (dashed green line). Pearson's correlation coefficients (R^2^) range from 0.994 to 0.918. HDV concentrations in IU/ml were calculated based on the linearity experiments using the WHO standard (10-fold dilution series; see Fig. 1A) and log-transformed. Gray dots mark the values considered outside of the linearity-range that were not included in the calculation of the slope. GT, genotype; HDV_UCT, HDV utility-channel; WHO, world health organization.

The LLOD of the HDV_UCT assay was determined as 3.86 IU/ml (95% CI 2.95–5.05 IU/ml) at a 95% detection probability with a linear range from 10^1^–10^8^IU/ml (GT1; Fig. 1A,B and Fig. S1). A linear relationship was observed for all 8 HDV GTs, with slopes ranging from -3.481 to -4.134 cycles/log and an R^2^ range from 0.918–0.994 (Fig. 2). Inter-run and intra-run variability were 0.3 and 0.6 Ct (3xLLOD), respectively, demonstrating a high assay reproducibility. No cross reactivity in patient serum samples containing a variety of bloodborne viral pathogens (n = 60) was observed. All samples of the qualitative EQA panel were identified correctly (16/16 positive, 4/4 negative). Viral loads in quantitative EQA panel samples available from 2020 (n = 4) were within the expected range (Fig. S1A). Comparison between results of the HDV_UCT and the CE-IVD assays showed that 58/110 and 49/110 samples were concordant positive or negative, respectively (Fig.1E). Three samples (all Ct: >35.6) only tested positive on the HDV_UCT assay (overall-agreement: 97.3%). Quantitative comparison demonstrated a strong correlation between assays (R^2^ 0.873; 95% CI 0.891–0.961; *p* value <0.0001; Fig. 1C). The Bland-Altman analysis revealed a mean of the bias of -0.2997 (95% CI 0.6914–1.291). Three datapoints appeared outside of the margins of ± 1.96 standard deviations of the mean (Fig. 1D). For HDV RNA-positive patient serum samples collected at different time points, both assays demonstrated similar viral load kinetics (Fig. S1B-D).

## Discussion

To improve the options for clinical HDV RNA monitoring, we established a new quantitative HDV RT-qPCR assay with excellent analytical performance and built-in full process control on the cobas6800 system. The use of highly automated, sample-to-result solutions for molecular diagnostics holds many inherent benefits over manual workflows, including improved reliability and reproducibility, as well as dynamic scaling of testing capacity. Moreover, the interpretation of test results is less error-prone since internal controls are included in every run and the quantitative test results are calculated using the Ct value by the laboratory information software based on the corresponding standard curve. Studies on assay performance on the cobas6800 already demonstrated high reliability for other viruses, including HBV, HCV and HIV^13^ and showed excellent comparability between testing sites.^14^ In line with previous studies,^15^ considerably less hands-on time (n = 24; HDV_UCT 20 min; CE-IVD 180 min) and manual steps (HDV_UCT 2; CE-IVD 36, without preparing reagents and data management) are required as nucleic acid extraction, purification, amplification, detection and data transfer to the laboratory information system are fully automated, hence minimizing the potential for errors.

The HDV_UCT assay showed excellent analytical and clinical performance. However, these promising results need to be further validated by examining a larger patient cohort. The new assay demonstrated an inclusivity for all 8 currently known GTs and a low limit of quantification of 10 IU/ml. Combined with the excellent comparability of the PCR results, the new assay will be advantageous for HDV screening and viral load monitoring during HDV treatment or clinical therapeutic trials.

## Financial support

The study was supported by 10.13039/100016545Roche Diagnostics. Marc Lütgehetmann, Maura Dandri, Dieter Glebe, Annika Giese, Nora Goldmann, Jan-Hendrick Bockmann and Julian Schulze zur Wiesch are funded by the 10.13039/100009139German Center for Infection Research (DZIF). Additional funding was provided by the German Research Foundation (DFG), SFB841 to Julian Schulze zur Wiesch, Marc Lütgehetmann, Maura Dandri and Lisa Sophie Pflüger; B08/SFB1021 and GL595/9-1 to Dieter Glebe. The National Reference Centre for Hepatitis B Viruses and Hepatitis D Viruses is supported by the German Ministry of Health via the Robert Koch Institute.

## Authors’ contributions

Lisa Sophie Pflüger, Marc Lütgehetmann: conceptualization, data curation, formal analysis, writing - original draft. Lisa Sophie Pflüger: visualization, investigation. Marc Lütgehetmann: supervision. Julian Schulze zur Wiesch, Dieter Glebe, Annika Giese, Nora Goldmann, Katja Giersch, Tassilo Volz, Maura Dandri, Jan-Hendrik Bockmann: resources. Dominik Nörz, Susanne Pfefferle, Julian Schulze zur Wiesch, Katja Giersch, Tassilo Volz, Maura Dandri: writing - reviewing and editing.

## Data availability statement

The data shown in this article are available from the corresponding authors upon a reasonable request. All reagents, antibodies and resources used in this research can be found in the CTAT table.

## Conflict of interest

Marc Lütgehetmann has received travel expenses and speakers’ honoraria (Roche Diagnostics). The other authors declare that they have no conflict of interest.

Please refer to the accompanying ICMJE disclosure forms for further details.
