# Supplementary material for: Clinical establishment of a laboratory developed quantitative HDV PCR assay on the cobas6800 high-throughput system
Source: JHEP Rep. 2021 Aug 28;3(6):100356. doi: 10.1016/j.jhepr.2021.100356 (PMC8531665; doi:10.1016/j.jhepr.2021.100356)
Supplement: Multimedia component 1 [file mmc1.pdf]

Clinical establishment of a laboratory developed quantitative HDV PCR assay on the cobas6800 high-throughput system

Lisa Sophie Pflüger, Dominik Nörz, Tassilo Volz, Katja Giersch, Annika Giese, Nora Goldmann, Dieter Glebe, Jan-Hendrik Bockmann, Susanne Pfefferle, Maura Dandri, Julian Schulze zur Wiesch, Marc Lütgehetmann

Table of contents

Fig. S1 2

A

EQA ID-number	Expected result – IU/ml	Range – IU/ml	HDV_UCT result – IU/ml
40041	5376	538 - 53760	2849
40042	2447	245 - 24470	1038
40043	8080	808 - 80800	3989
40044	17543	1754 - 1755430	15320

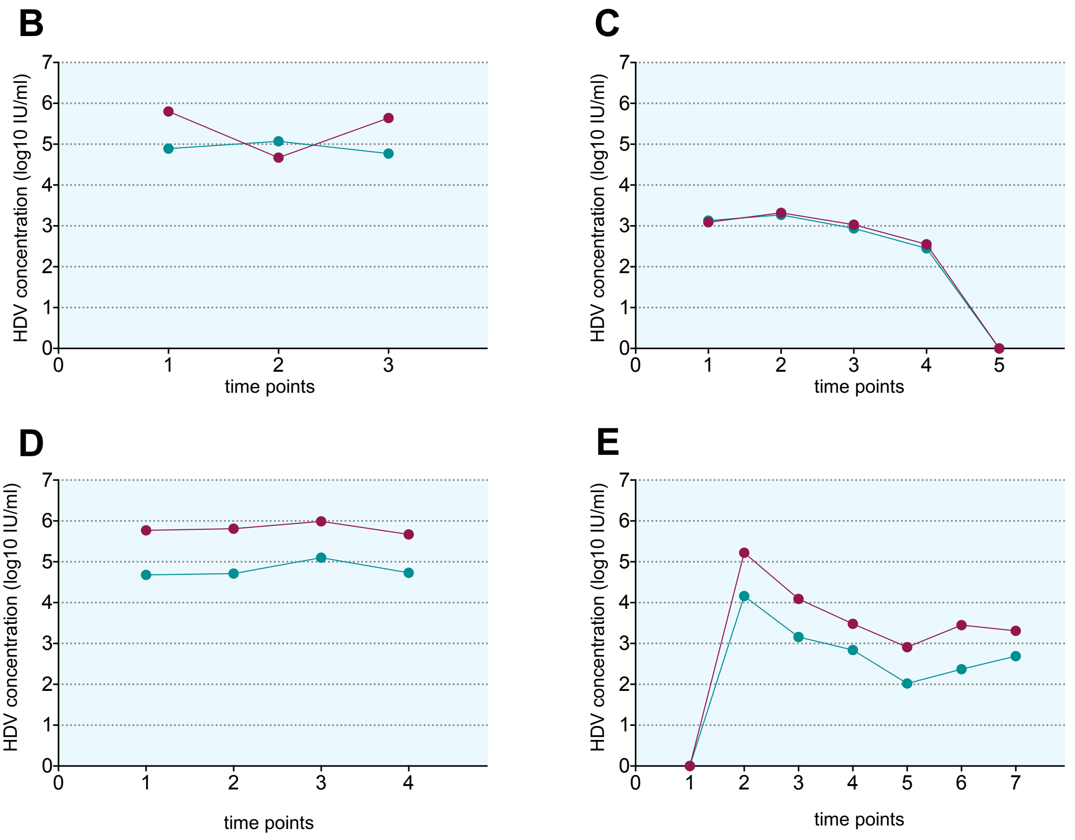

Fig. S1. Test results of the EQA panel from 2020 and of the comparison of the HDV_UCT and CE-IVD assay test results for four different HDV-RNA+ patients tested at different time points.

(A) Test results of the HDV_UCT assay and the corresponding expected values including the range for the EQA panel samples available from 2020 are displayed. (B-E) To demonstrate assay performance over the disease course serum samples collected at different time points from four different HDV-RNA positive patients were included and examined using the HDV_UCT (violet dots) and the CE-IVD assay (green dots). Both assays show a similar kinetic of viral load over time. The quantitative values vary up to one log. Patient 2 (C) underwent treatment with PEGylated interferon alpha and patient 4 (E) was diagnosed with an acute HDV infection.

Abbreviations: CE-IVD, CE-marked in vitro diagnostics assay; EQA, external quality assessment; HDV_UCT, hepatitis delta virus utility channel assay.
