# Supplementary material for: Clinical establishment of a laboratory developed quantitative HDV PCR assay on the cobas6800 high-throughput system
Source: JHEP Rep. 2021 Aug 28;3(6):100356. doi: 10.1016/j.jhepr.2021.100356 (PMC8531665; doi:10.1016/j.jhepr.2021.100356)
Supplement: Multimedia component 2 [file mmc2.docx]

**JHEP Reports**

**CTAT methods**

Tables for a “Complete, Transparent, Accurate and Timely account” (CTAT) are now mandatory for all revised submissions. The aim is to enhance the reproducibility of methods.

- Only include the parts relevant to your study
- Refer to the CTAT in the main text as ‘Supplementary CTAT Table’
- Do not add subheadings
- Add as many rows as needed to include all information
- Only include one item per row

**If the CTAT form is not relevant to your study, please outline the reasons why:**

|  |
| --- |

- 1. **Antibodies**

| **Name** | **Citation** | **Supplier** | **Cat no.** | **Clone no.** |
| --- | --- | --- | --- | --- |
|  |  |  |  |  |

- 1. **Cell lines**

| **Name** | **Citation** | **Supplier** | **Cat no.** | **Passage no.** | **Authentication test method** |
| --- | --- | --- | --- | --- | --- |
|  |  |  |  |  |  |

- 1. **Organisms**

| **Name** | **Citation** | **Supplier** | **Strain** | **Sex** | **Age** | **Overall n number** |
| --- | --- | --- | --- | --- | --- | --- |
|  |  |  |  |  |  |  |

- 1. **Sequence based reagents**

| **Name** | **Sequence** | **Supplier** |
| --- | --- | --- |
| Forward primer | CTCCCTTWGCCATCCmGAG | IDT DNA Technologies |
| Reverse primer | CTCTTCGGGTCGGCATmGG- | IDT DNA Technologies |
| Probe | FAM-ATGCCCAGGTCGGACCRC-MGB | biomers.net GmbH |
|  | m= 2’-O-methyl RNA bases |  |

- 1. **Biological samples**

| **Description** | **Source** | **Identifier** |
| --- | --- | --- |
| HDV GT 1 | Cell culture virus | Hamburg (AJ000558) |
| HDV GT 2 | Cell culture virus | Hamburg (MG557658) |
| HDV GT 3 | Cell culture virus | Hamburg (L22063) |
| HDV GT 4 | Cell culture virus | Hamburg (AF209859) |
| HDV GT 5 | Cell culture virus | AG Glebe (GT5) |
| HDV GT 6 | Cell culture virus | AG Glebe (GT6) |
| HDV GT 7 | Cell culture virus | AG Glebe (GT7) |
| HDV GT 8 | Cell culture virus | AG Glebe (GT8) |
| HDV 1^st^ WHO standard | Paul-Ehrlich-Institut | 7657/12 |

- 1. **Deposited data**

| **Name of repository** | **Identifier** | **Link** |
| --- | --- | --- |
|  |  |  |

- 1. **Software**

| **Software name** | **Manufacturer** | **Version** |
| --- | --- | --- |
| BioRender | BioRender |  |
| GraphPad Prism | Graphpad Software, Inc | version 8 |

- 1. **Other (*e.g*. drugs, proteins, vectors etc.)**

|  |  |  |
| --- | --- | --- |
|  |  |  |

- 1. **Please provide the details of the corresponding methods author for the manuscript:**

| Dr. med. Marc Lütgehetmann,  University Medical Center Hamburg-Eppendorf (UKE),  Center for Diagnostics,  Institute of Medical Microbiology, Virology and Hygiene,  026, 2.OG, R 232  Martinistraße 52  20246 Hamburg  Fax: +49741054881  Tel: +49741051772  Email: mluetgeh@uke.de |
| --- |
|  |

**2.0 Please confirm for randomised controlled trials all versions of the clinical protocol are included in the submission. These will be published online as supplementary information.**

| **NA** |
| --- |
